# Structural Damage Early Warning Method of Quayside Container Crane Based on Fuzzy Entropy Ratio Variation Deviation

**DOI:** 10.3390/s24237575

**Published:** 2024-11-27

**Authors:** Jiahui Liu, Jian Zhao, Dong Zhao, Xianrong Qin

**Affiliations:** 1 School of Technology, Beijing Forestry University, Beijing 100083, China; zhaojian1987@bjfu.edu.cn (J.Z.); zhaodong68@bjfu.edu.cn (D.Z.); 2 School of Mechanical Engineering, Tongji University, Shanghai 201804, China; tjqin@tongji.edu.cn

**Keywords:** structural health monitoring, dual-tree complex wavelet transform, fuzzy entropy, stiffness reduction, damage identification, damage early warning, quayside container crane

## Abstract

Real-time monitoring and early warning of structures are essential for assessing structural health and ensuring safety maintenance. To improve the timeliness of early warnings for structural abnormal states in quayside container cranes (QCCs) with incomplete damage data, a structural abnormal state early warning method based on fuzzy entropy ratio variation deviation (FERVD) is proposed. First, monitoring data are subjected to dual-tree complex wavelet transform (DTCWT). The adaptive frequency bands obtained from the decomposition, combined with fuzzy entropy (FE), are used to extract response signal features and construct the FERVD warning indicator. Based on this indicator, dynamic thresholds for early warning are established to differentiate between structural health states and various damage conditions. Secondly, a finite element model of structure for QCCs is developed. By simulating damage at various locations and severities through the stiffness reduction of different elements, a comprehensive structural simulation monitoring dataset is generated. The efficacy of the proposed early warning method is validated through numerical experiments and engineering case studies. The numerical results demonstrate that the proposed method effectively distinguishes between different damage conditions and provides timely warnings for various damage states. Furthermore, engineering case analysis shows that when the structure is in a healthy state, the FERVD values at different monitoring points fluctuate within the threshold range, indicating the applicability of the proposed method in the structural health monitoring (SHM) of QCCs.

## 1. Introduction

Engineering structures are susceptible to human-induced and environmental factors which accelerate the accumulation of damage and shorten the structural life cycle [1,2]. Structural health monitoring (SHM) and damage warning have emerged as critical topics across various disciplines, including civil engineering, mechanical engineering, materials, electrical, aerospace and other fields [3,4,5]. Especially in the field of civil engineering, large-scale engineering structures, such as bridges, truss buildings and long-span spatial structures, can have service lives extending over several decades or even centuries. The effect of environmental erosion, material aging, long-term loading, fatigue and mutation will inevitably contribute to the damage accumulation and performance degradation of structures, potentially leading to accidents and casualties. Compared with large bridges, super high-rise buildings and other civil structures, engineering machinery structures exhibit greater complexity. Damage tends to accumulate over time, resulting in an increase in both the number and severity of damage locations. SHM enables the assessment of current health states, facilitates early warnings to prevent catastrophic failures, and provides essential strategies for maintenance [6,7,8,9]. To effectively capture the health status and performance degradation of structures in time, it is crucial to implement damage detection and early warning for engineering structures, prompting the identification of damage locations and severity.

Structural responses can reflect changes in health conditions; specifically, structural damage alters physical and modal parameters, leading to changes in the dynamic response of the structure. The occurrence, location, and severity of damage can be inferred from the variations in structural modal parameters before and after damage. However, in practical engineering applications, the changes in inherent dynamic characteristics due to damage are often minimal and susceptible to noise, rendering them unsuitable as parameters for assessing structural damage states [10,11]. Therefore, it is necessary to identify dynamic characteristic indices that exhibit greater sensitivity to damage and robustness against noise. Consequently, research in structural damage diagnosis increasingly emphasizes the establishment of methods for structural abnormal state early warning and damage identification based on these indices.

Currently, damage detection techniques based on vibration data have been extensively studied, utilizing measured changes in the dynamic features of structures to indicate structural damage [12,13]. Structural abnormal state early warning constitutes a significant objective of damage detection efforts. Pan et al. proposed a novel structural health monitoring method that employs wavelet packet energy spectrum analysis to identify structural damage in real time and facilitate early warnings [14]. Similarly, Ni et al. utilized a Bayesian regression model combined with the reliability theory to formulate an anomaly index for evaluating the health condition of bridge expansion joints, providing damage warnings once the damage exceeded a specified threshold [15]. Additionally, Ref. [16] introduced a dynamic early warning model for rockbursts using microseismic multi-parameter data based on a Bayesian network. Furthermore, Yang et al. developed an innovative early warning method for the longitudinal tearing of conveyor belts through infrared spectrum analysis [17]. Refs. [18,19,20,21] proposed the use of machine learning and probabilistic statistical methods to establish early warning models based on structural or condition monitoring data, enabling timely warnings for the monitoring of objects in various fields.

Establishing a reasonable early warning threshold is a critical issue in structural abnormal state early warning. The early warning threshold defines the safety limits of structures, and the appropriateness of the threshold directly impacts the timeliness and reliability of the warnings. If the threshold is set too stringently, the system may generate frequent false alarms; conversely, if it is set too leniently, there is a risk of overlooking or omitting critical hidden dangers that warrant attention. Xie et al. proposed an early warning threshold framework for the risk of subway structure collapse, utilizing the D-S evidence theory to integrate primary and secondary data fusion based on the rough set theory, thereby enabling dynamic predictions of collapse risk trends during subway construction [22]. Rafei et al. introduced a warning threshold for monitoring tuberculosis incidence rates, constructing multiple hypothesis thresholds based on an autoregressive model estimation and revealing the optimal threshold through ROC curve analysis [23]. Wang et al. presented an early warning mechanism and associated thresholds for structural responses based on statistical and extreme value analyses of historical timber buildings, realizing early warnings for substructures under varying data accumulation times [24]. Despite advancements in warning threshold research, there remains a lack of clear definitions for warning indicators, leading to uncertainty in their selection. The appropriate selection of characteristic indicators is pivotal for the accuracy of the early warning threshold, which in turn affects the timeliness and precision of the warnings [25]. Therefore, further research is needed to identify suitable early warning indices and establish effective early warning thresholds to enhance the accuracy of structural abnormal warnings.

In complex engineering structures, many dynamic parameters are often insensitive to early signs of structural abnormalities, making it challenging to assess the severity of such damage. Furthermore, the presence of uncertain factors, such as noise, significantly influences the selection of appropriate damage characteristic parameters, which in turn impacts the accuracy of structural damage warnings [26]. Generally, the methods of setting damage indices are different, and there is no standardized approach to determine the reasonableness of warning indicators for structural damage. However, a well-defined indicator set can facilitate timely early warnings for structural abnormalities, demonstrating superior effectiveness, compared to traditional modal parameters used as early warning indicators, when evaluating the timeliness and accuracy of alerts.

Most of the previous research on structural abnormal state early warning has primarily focused on identifying whether damage has occurred and issuing alarms accordingly. There is a notable lack of studies addressing hierarchical early warnings based on the severity of damage. Additionally, the choice of warning indicators and thresholds plays a crucial role in the accuracy of damage detection and the timeliness of early warnings. Further investigation is essential to enhance the reliability of damage assessments.

Therefore, this paper proposes a novel approach for early damage warning: damage features are extracted using adaptive frequency band fuzzy entropy derived from dual-tree complex wavelet transform, serving as early warning indicators for detecting the types of structural damage. A baseline warning threshold is established for varying severities of damage based on the dynamic changes in the indicators between normal and abnormal states. The effectiveness of the proposed early warning methods is demonstrated through a case study involving typical structural damage data from QCCs. The research on early warning methods is instrumental in detecting and providing early warning for potential structural damage prior to failures.

## 2. Methods

**Figure 1:**
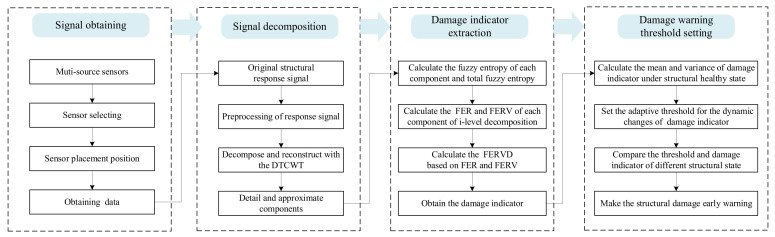
The flowchart of the structural damage early warning approach.

In the context of structural damage early warning, the effectiveness of early warning depends on two critical factors: the selection of early warning indicators and the establishment of warning thresholds. To enable the timely detection of structural damage, reduce false alarms, and improve the accuracy of early warnings, a novel early warning indicator, termed the fuzzy entropy ratio variation deviation (FERVD), with an associated early warning method are proposed. The process of damage early warning is shown in Figure 1.

### 2.1. Fuzzy Entropy (FE)

Fuzzy entropy (FE), which has a similar physical significance to sample entropy (SE), quantifies the complexity and irregularity of time series data. Fuzzy entropy employs the concept of fuzzy functions, using exponential fuzzy functions to calculate the similarity between two vectors. This method overcomes the limitations of sample entropy, which relies on the Heaviside function and may not accurately reflect the actual boundaries of sample classes, enhancing the robustness of time series analysis against noise [27,28,29].

The calculation steps for fuzzy entropy (FE) can be described as follows [30,31].

Step 1. $\left\{x(i)\right\}=\left\{x(1),x(2),\ldots ,x(N)\right\}$ is a time series of length N, the elements of $\left\{x(i)\right\}$ are organized into m dimensional vectors $\left\{x(1),x(2),\ldots ,x(N-m+1)\right\}$, represented as follows:

(1) $$\left\{\begin{array}{l} X_{i}^{m}=\left\{x(i),x(i+1),\ldots ,x(i+m-1)\right\}-x_{0}(i),1\leq i\leq N-m+1\\ x_{0}(i)=\frac{1}{m}\sum_{j=0}^{m-1}x(i+j) \end{array}\right.$$

where $x_{0}(i)$ is the mean of $X_{i}^{m}$, and $X_{i}^{m}$ is determined after removing the mean $x_{0}(i)$ from time series.

Step 2. Calculate the maximum distance $d\left[X_{i}^{m},X_{j}^{m}\right]$ between $X_{i}^{m}$ and $X_{j}^{m}$, represented as follows:

(2) $$d_{ij}^{m}=d\left[X_{i}^{m},X_{j}^{m}\right]=\max_{k=0,\ldots ,m-1}(\left|\left(x(i+k)-x_{0}(i))-(x(j+k)-x_{0}(i)\right)\right|),i,j=1,2,\ldots ,N-m;i\neq j$$

Step 3. The similarity $D_{ij}^{m}$ of vectors $X_{i}^{m}$ and $X_{j}^{m}$ is defined by a fuzzy function $\mu (d_{ij}^{m},n,r)$, given by

(3) $$D_{ij}^{m}=\mu (d_{ij}^{m},n,r)=e^{-{\left(d_{ij}^{m}/r\right)}^{n}}$$

where $\mu (d_{ij}^{m},n,r)$ is the exponential function, and n and r are the gradient and width of the boundary, respectively.

Step 4. Define the function $\varphi^{m}(n,r)$ in the following:

(4) $$\varphi^{m}(n,r)=\frac{1}{N-m}\sum_{i=1}^{N-m}(\frac{1}{N-m-1}\sum_{j=1,j\neq i}^{N-m}D_{ij}^{m})$$

Step 5. Similarly, for m+1 dimension, repeat Equations (2)–(4), the function is denoted as

(5) $$\varphi^{m+1}(n,r)=\frac{1}{N-m}\sum_{i=1}^{N-m}(\frac{1}{N-m-1}\sum_{j=1,j\neq i}^{N-m}D_{ij}^{m+1})$$

Step 6. The fuzzy entropy is defined as

(6) $$FuzzyEn(m,n,r)=\underset{N\to \infty }{\lim}\left[\ln\varphi^{m}(n,r)-\ln\varphi^{m+1}(n,r)\right]$$

Step 7. When the length of data N is finite, the Formula (7) is expressed as

(7) $$FuzzyEn(m,n,r,N)=\underset{N\to \infty }{\lim}\left[\ln\varphi^{m}(n,r)-\ln\varphi^{m+1}(n,r)\right]$$

According to the definition of fuzzy entropy, its result is influenced by several parameters: the embedding dimension m, the similarity tolerance r, the gradient of the fuzzy function n, and the length of the data N. The length of the data N has minimal impact on the fuzzy entropy result. For the embedding dimension m, a larger value allows for the reconstruction of the joint probability of the time series with more detailed information; however, an excessively large m can be detrimental due to it requiring longer data, which may be impractical. Concerning the similarity tolerance r, if r is too large, significant statistical information may be lost. Conversely, if it is too small, the estimation of statistical characteristics may be suboptimal, making the result more sensitive to noise. The gradient n is also crucial in calculating the similarity between vectors for fuzzy entropy. If n is too large, detailed information may be lost. Generally, it is advisable to consider n as a smaller integer value in calculations. Therefore, selecting appropriate parameters is essential for accurately calculating fuzzy entropy, as it directly affects the results obtained.

### 2.2. Construction of FERVD Damage Warning Indicators

According to Reference [28], nonlinear analysis methods based on entropy have been effectively applied to damage feature extraction. This paper proposes the concept of dual-tree complex wavelet fuzzy entropy, which is grounded in fuzzy entropy, to evaluate the complexity of time series across different frequency bands. In contrast to multi-scale fuzzy entropy, wavelet fuzzy entropy is capable of capturing the complexities of both high-frequency and low-frequency components. To facilitate a more comprehensive analysis of nonlinear and non-stationary signals within each frequency band, dual-tree complex wavelet fuzzy entropy is employed as a damage feature and warning indicator for structural damage. The process for extracting damage warning indicators is as follows.

(1) The structural dynamic response k(n) (n=1,2,…,N), n is the number of sampling points) is decomposed using the i-level dual-tree complex wavelet, and $k_{i}$ is the structural dynamic response at the i-level decomposition.

(2) The fuzzy entropy of structural dynamic response $k_{i}$ in each component of i-level decomposition can reflect the dynamic characteristics of the structure. The total fuzzy entropy for each component at each decomposition level is calculated using Equations (7) and (8):

(8) $$FE_{total}=\sum_{i=1}^{m}FE_{i}$$

where i is the decomposition level, m is the maximum decomposition level, and $FE_{i}$ represents the fuzzy entropy of the component at the i-level decomposition.

(3) The fuzzy entropy ratio (FER) of each component is used as the structural damage early warning parameter vector

(9) $$FER_{i}=\frac{FE_{i}}{FE_{total}}(i=1,2,\ldots ,m)$$

The structural damage is determined through the fuzzy entropy ratio variation (FERV)

(10) $$FERV_{i}=\left|FER_{hi}-FER_{di}\right|(i=1,2,\ldots ,m)$$

where $FERV_{i}$ denotes the fuzzy entropy ratio variation of the i-level component, and $FER_{hi}$ and $FER_{di}$ represent the i-level fuzzy entropy ratio for structural healthy and damaged states, respectively.

(4) On the basis of FERV, a novel structural damage warning indicator, called fuzzy entropy ratio variation deviation (FERVD), is defined based on the dynamic change characteristics of the fuzzy entropy in each component of DTCWT

(11) $$FERVD=\sqrt{\sum_{i=1}^{m}(FERV_{i}-\bar{FERV}}{)}^{2}$$

where $\bar{FERV}$ is the mean of $FERV_{i}$.

### 2.3. Setting of FERVD Warning Threshold

The setting an appropriate threshold is crucial for effective structural damage early warning. In practical engineering applications, traditional thresholds are typically established based on engineering experience or the statistical probability distribution of monitoring data from structures in a healthy state, often resulting in a fixed threshold. However, fuzzy entropy determined by the dual-tree complex wavelet transform (DTCWT), varies with the structural state, leading to corresponding fluctuations in both the damage warning indicator and the baseline threshold. To accommodate these variations, the early warning threshold must be adaptively updated in response to changes in the indicator. According to References [32,33], the normal reference value of the indicator for the structural healthy state is used as the threshold, with the mean and standard deviation of the warning indicator being adaptively updated as described in the Equation (12).

(12) $$\left\{\begin{array}{l} TL_{u}=\mu_{FERVD_{n}}+\sqrt{\sigma_{FERVD_{n}}}\\ TL_{d}=\mu_{FERVD_{n}}-\sqrt{\sigma_{FERVD_{n}}} \end{array}\right.$$

where $\mu_{FERVD_{n}}$ is the mean of the warning indicator under structural healthy state, $\sigma_{FERVD_{n}}$ is the variance of warning indicator under structural healthy state, and $TL_{u}$ and $TL_{d}$ are the upper threshold and lower threshold, respectively. Through comparing whether the dynamic changes in damage warning indicator exceed the threshold, it is possible to provide an early warning for potential structural damage. If the warning indicator exceeds the threshold, it indicates that structural damage is occurring, triggering timely alarms.

## 3. Numerical Example

### 3.1. Damage Simulation of Finite Element Model for Structure of QCCs

**Figure 2:**
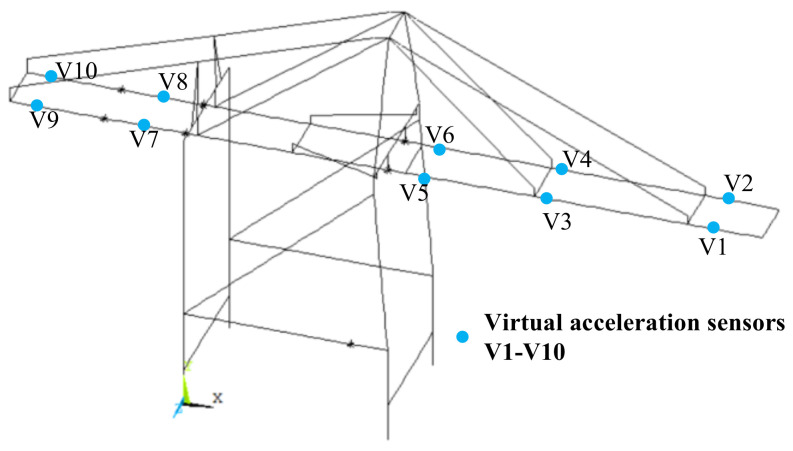
The locations of sensor placement for each monitoring point of a QCC.

A finite element model of the structure for a QCC has been established, incorporating virtual sensors to monitor dynamic responses at various locations within the structure. A statistical investigation of metal structure failures in QCCs revealed that major faults or damages frequently occur in the main beam and forestry. Consequently, vibration monitoring points were strategically placed at critical locations susceptible to damage in both the front and rear beams. Virtual accelerometers were positioned at areas characterized by intense vibrations or significant modes of vibration, including the maximum forward extension of the front beam, the connection between the front beam and the middle forestry, the front beam near the sea-side door frame, the mid-span of the rear beam, and the maximum backward extension of the rear beam, totaling 10 monitoring points. The locations of sensor placement are illustrated in Figure 2, while the monitoring directions for each monitoring point are detailed in Table 1.

**Figure 3:**
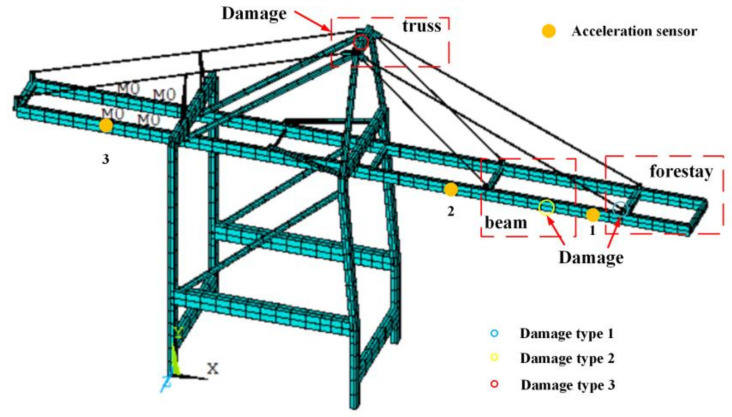
Three types of damage for structures of QCCs.

In the structural finite element model of a QCC, different severities of damage were introduced at various structural locations, specifically at the mid-span of the front beam, the top of the truss, and the end of the forestay. Three types of damage are illustrated in Figure 3. The degree of damage was quantified by applying a stiffness reduction to specific elements, with each damage location exhibiting seven levels of severity, ranging from 5% to 35%. Consequently, the constructed simulation dataset encompassed a total of 21 damage conditions and one undamaged condition, as summarized in Table 2.

During operational conditions, the rated load of the trolley is 65 tons. The trolley lifts a fully loaded cargo from the maximum forward extension of the front beam and moves at a constant speed to the midpoint of the rear beam, where it unloads the cargo before returning empty at the same constant speed. In order to approach the actual operating conditions of QCCs more closely, a moving mass load is used as a random excitation of the main beam in the finite element model, effectively simulating the operational process of the trolley on the beam and acquiring acceleration response data at each monitoring point under both undamaged and various damaged conditions. For each damage condition, the virtual accelerometers at the monitoring points were sampled at a frequency of 100 Hz over a duration of 72 s, constructing a simulation dataset for each monitoring point that contains 7200 data points in different conditions.

### 3.2. Results and Discussion

**Figure 4:**
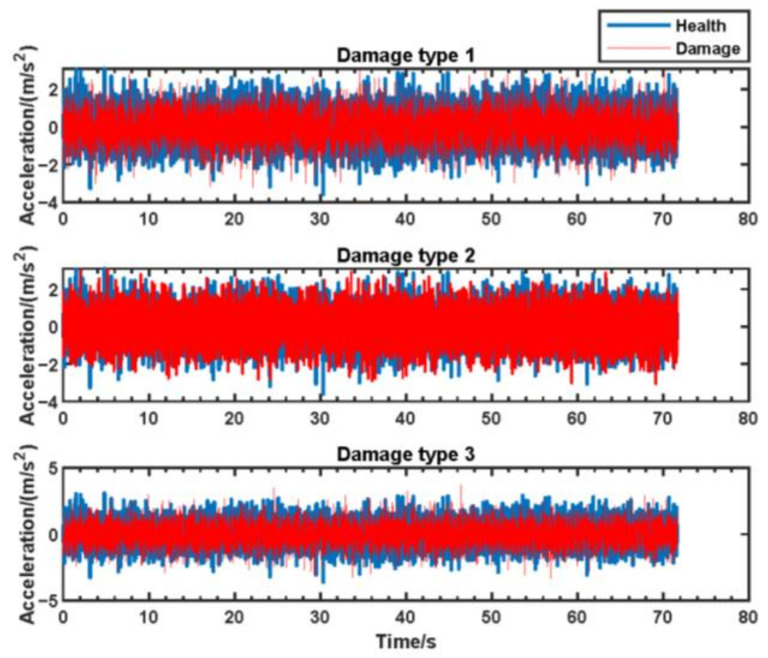
The acceleration responses for monitoring point 1.

The acceleration responses for monitoring points 1, 2, and 3 (as illustrated in Figure 2) were extracted from the constructed simulation dataset under various damage conditions. The acceleration response for monitoring point 1 is shown in Figure 4.

To demonstrate the sensitivity and noise robustness of the FERVD indicators, data samples for three types of structural damage obtained from monitoring point 1 were preprocessed. According to the damage early warning procedure in Section 2, a dual-tree complex wavelet decomposition with four layers was applied to the preprocessed data samples to extract the FERVD indicators.

**Figure 5:**
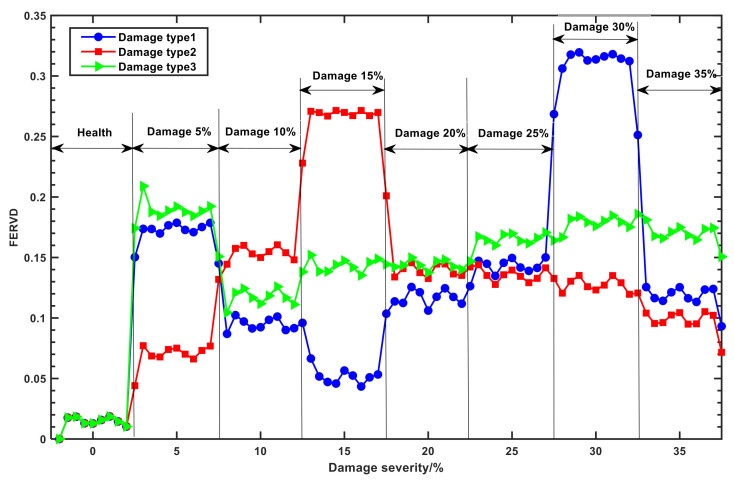
The variations of the FERVD indicators at monitoring point 1.

Figure 5 illustrates the variations of the FERVD indicators at monitoring point 1. As depicted in Figure 5, under healthy conditions, the FERVD indicators for the end of the forestay, the middle of the front beam, and the top of the truss exhibit similar trends and fluctuate within a narrow range, making it difficult to distinguish among different elements. However, while the severity of damage increases, deviation from the healthy state begins to occur in the FERVD indicators, showing a clearer differentiation of damage at the end of the forestay, the middle of the front beam, and the top of the truss.

**Figure 6:**
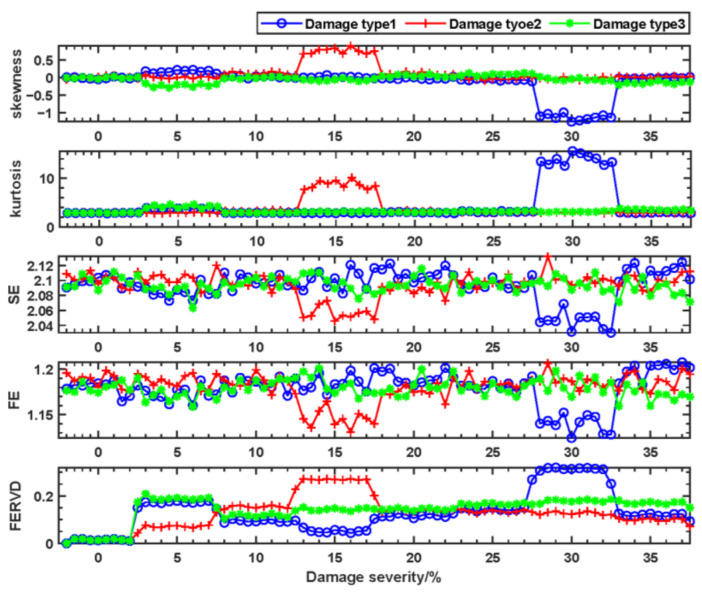
The comparison of time-domain feature indicators and FERVD indicators at monitoring point 1 without preprocessing.

Figure 6 shows a comparison of time-domain feature indicators and FERVD indicators at monitoring point 1 without preprocessing. The results indicate that noise partially obscures the variations in indicators for the end of the forestay, the middle of the front beam, and the top of the truss. Although skewness and kurtosis exhibit some robustness against noise, they are insufficient for effectively differentiating damage among the aforementioned elements under varying damage severities. In contrast, indicators such as SE and FE demonstrate reduced robustness to noise compared to skewness and kurtosis, and they also fail to distinguish between different damaged elements at varying severities. Notably, the FERVD indicator shows relatively better robustness against noise compared to the other feature indicators. Under different damage conditions, it effectively distinguishes damage at the end of the forestay, the middle of the front beam, and the top of the truss, indicating that the FERVD indicator is sensitive to structural damage.

**Figure 7:**
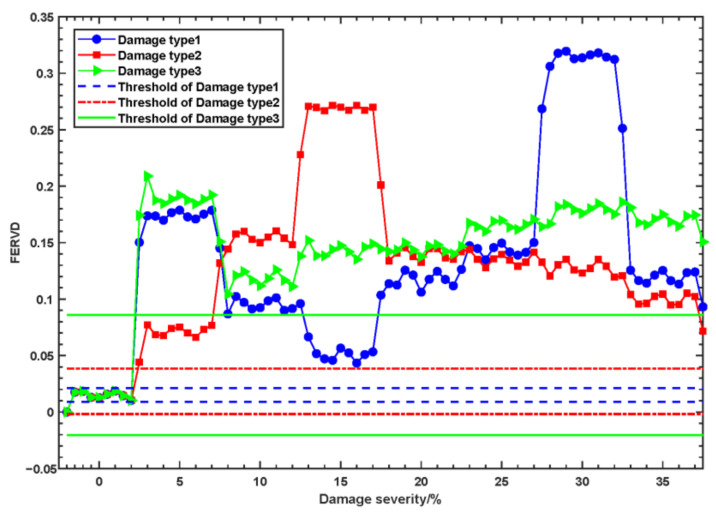
The FERVD warning thresholds vary among different damaged elements.

According to the early warning threshold setting method introduced in Section 2.3, the FERVD indicator warning thresholds for different damage types and severities at monitoring point 1 were determined, as shown in Figure 7. Figure 7 demonstrates that the FERVD warning thresholds vary among different damaged elements. The FERVD warning thresholds of damage type 1 is [0.0097~0.0236]; the FERVD warning thresholds of damage type 2 is [−0.003~0.0398]; the FERVD warning thresholds of damage type 3 is [−0.02~0.0875]. When the FERVD indicators fluctuate within the threshold range, all structural elements remain in a healthy state. However, when damage occurs in different elements, the FERVD values gradually exceed the threshold in correlation with increasing damage severity.

**Figure 8:**
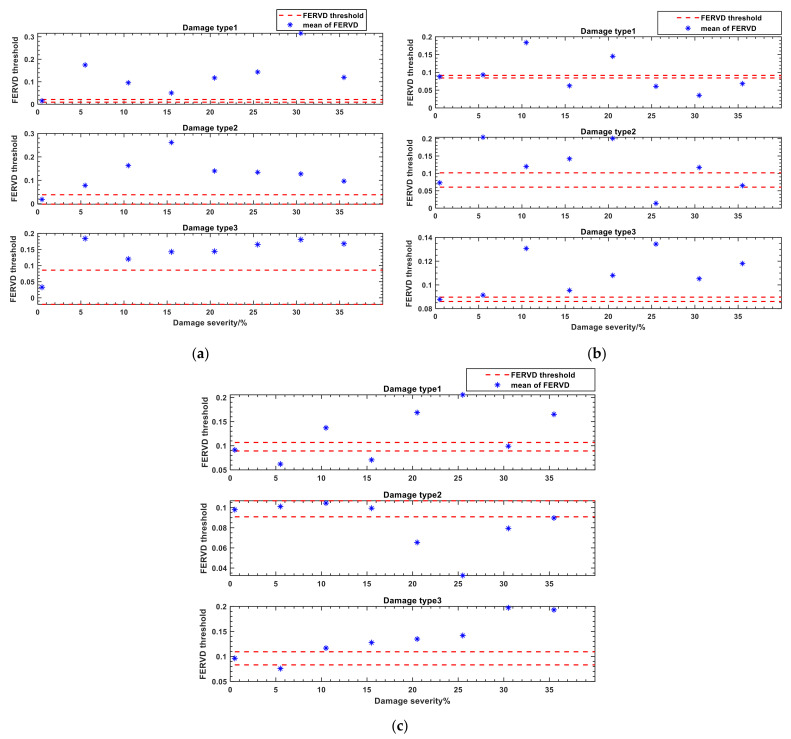
The warning results for monitoring points 1, 2, and 3 under various damage types and severities: (**a**) The warning results for monitoring point 1; (**b**) The warning results for monitoring point 2; (**c**) The warning results for monitoring point 3.

To compare the early warning effectiveness of different monitoring points, Figure 8 displays the warning results for monitoring points 1, 2, and 3 under various damage types and severities. As shown in Figure 8, the FERVD warning thresholds of different damage locations for different monitoring points are not the same. The FERVD mean values at monitoring points 1, 2, and 3 all remain within the threshold range, indicating that there is no damage (damage severity is 0%) at the end of the forestay, the middle of the front beam, and the top of the truss. It suggests that the structure is in a healthy state, and no warning is issued under this condition.

As illustrated in Figure 8a, at monitoring point 1, the FERVD mean values fall outside the thresholds for various damage severities occurring at the end of the forestay, the middle of the front beam, or the top of the truss. It indicates that the FERVD indicator at monitoring point 1 can effectively provide early warnings for different damaged elements.

In Figure 8b, at monitoring point 2, the FERVD mean values gradually exceed the thresholds with damage severity increases at the end of the forestay, the middle of the front beam, or the top of the truss. However, when the damage severity at the top of the truss reaches 35%, the FERVD mean values fall within the threshold range. Consequently, the damage at the top of the truss does not trigger a warning despite exceeding the threshold, indicating a discrepancy in the results. Therefore, the FERVD indicator at monitoring point 2 did not fully provide accurate warnings for different damaged elements.

Figure 8c shows that at monitoring point 3, the FERVD mean values begin to exceed the threshold only when the damage severity at the end of the forestay reaches 20%. Under this severity, the FERVD mean values remain within the threshold and are considered as indicating a healthy state. It reflects that monitoring point 3 is not sensitive to early damage at the end of the forestay. Furthermore, when the damage severity at the middle of the front beam reaches 30%, the FERVD mean value falls within the threshold, not exceeding the threshold despite occurring the damage, leading to a warning deviation.

Thus, the effectiveness of the FERVD indicator for early warning varies among different monitoring points. Monitoring points that are closer to the damaged elements provide more accurate warning results when damage occurs in the structure.

## 4. Case Study in Engineering

**Figure 9:**
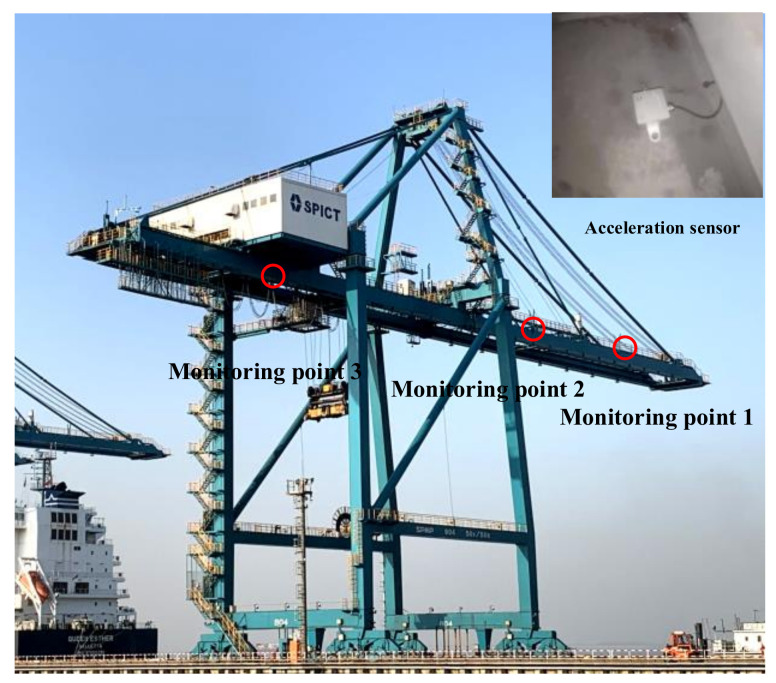
Location of monitoring points for structure of a QCC.

To evaluate the applicability and effectiveness of the proposed structural anomaly early warning method in practical engineering, this section focuses on a QCC at a certain port as the monitoring subject. The monitoring data collected from actual monitoring points, corresponding to the locations used in the numerical experiments, are utilized to illustrate the early warning capabilities. Monitoring point 1 is located at the maximum forward extension of the front beam, monitoring point 2 is located at the front beam near the seaward door frame, and monitoring point 3 is at the mid-span of the rear beam, as shown in Figure 9.

**Figure 10:**
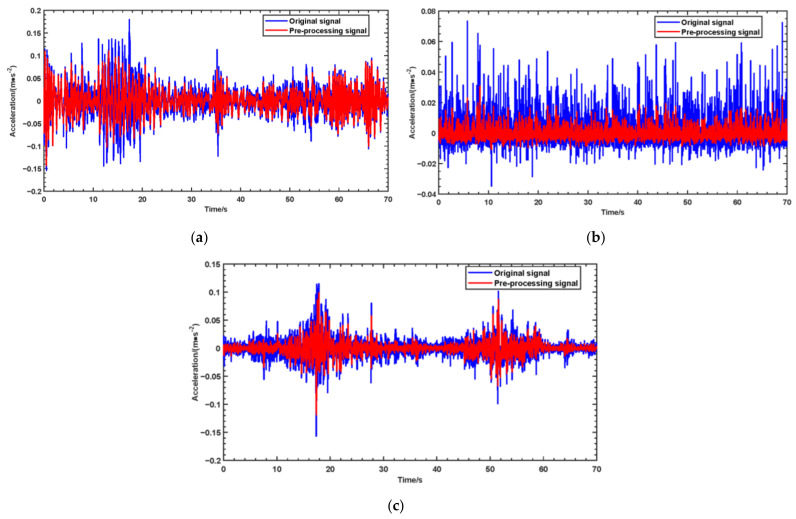
The original signal and the preprocessed monitoring signal collected from monitoring point 1, 2 and 3 under the operational conditions of QCCs: (**a**) Monitoring point 1; (**b**) Monitoring point 2; (**c**) Monitoring point 3.

To validate the proposed method, monitoring point 1 is used as an example. Figure 10 displays the original signal and the preprocessed monitoring signal collected from different monitoring points under the operational conditions of the QCC. The monitoring data obtained from the aforementioned monitoring points represent the structural health status of the QCC; the early warning indicator calculation methods and threshold setting approaches described in Section 2.2 and Section 2.3 can be applied accordingly. The FERVD indicators and their corresponding thresholds for different monitoring locations within the specified duration are calculated, with a calculation window width set to 200 (equivalent to a duration of 2 s).

**Figure 11:**
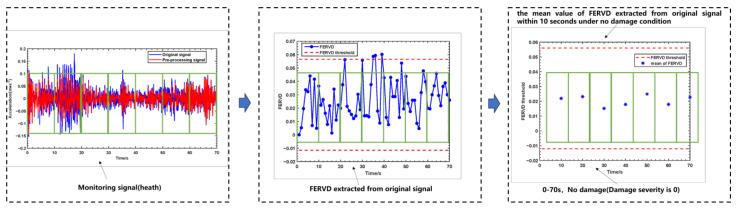
The variations of the FERVD indicator at monitoring point 1 over the specified duration and the early warning results of FERVD over different time periods for monitoring point 1.

Figure 11 illustrates the variations of the FERVD indicator at monitoring point 1 over the specified duration, along with the early warning results of FERVD across different time segments. It can be observed from Figure 11 that there are instances where individual FERVD values exceed the threshold, while the majority of FERVD values fluctuate within the threshold range. To minimize the influence of such random factors, the mean FERVD values for each monitoring point are calculated at 10 s intervals. As is shown in Figure 11, by comparing the mean FERVD values with the corresponding thresholds, it is evident that the mean FERVD values extracted from the original signal within 10 s intervals for monitoring point 1 remain within the threshold, indicating that no structural damage has occurred. Due the obtained monitoring data reflecting a healthy state, the early warning results for monitoring point 1 are consistent with the actual structural conditions.

Table 3 presents the early warning results of the FERVD indicators for different monitoring points across various time segments. It can be observed from Table 3 that the mean FERVD values for different monitoring points and time periods remain within the respective threshold ranges. This indicates that the structure of the QCC is in a healthy condition.

## 5. Conclusions

(1) This study introduces a structural anomaly early warning indicator based on the adaptive frequency band of dual-tree complex wavelet decomposition, combined with fuzzy entropy, along with its corresponding early warning method. The effectiveness of this early warning method has been experimentally validated using both simulated data and engineering monitoring data.

(2) Numerical experiments and comparative analyses demonstrate that the proposed FERVD early warning indicator is sensitive to damage and can effectively distinguish between different damage conditions. The setting of the warning threshold facilitates the early detection of various structural damage states to a certain severity; however, the warning effectiveness varies among different monitoring points, with those located closer to the damage exhibiting more accurate results. At monitoring point 2, when the damage severity at the top of the truss reaches 35%, the FERVD mean values fall within the threshold range; at monitoring point 3, when the damage severity at the middle of the front beam reaches 30%, the FERVD mean value falls within the threshold, not exceeding the threshold despite occurring the damage, leading to a warning deviation.

(3) The results from engineering case analyses indicate that when the structure is in a healthy state without any damage, the FERVD values at various monitoring points fluctuate within the threshold range. This early warning method also demonstrates a severity of applicability in the monitoring of structures for QCCs.

## Figures and Tables

**Table 1:** Monitoring direction of each monitoring point for a QCC.

Sensors	Monitoring Sites	Monitoring Directions
V1, V2	the maximum forward extension of the front beam	X, Y
V3, V4	the connection between the front beam and the middle forestry	X, Z
V5, V6	the front beam near the sea-side door frame	X, Y
V7, V8	the mid-span of the rear beam	X, Y
V9, V10	the maximum backward extension of the rear beam	X, Y

**Table 2:** Description of each damage condition for QCCs.

Damaged Type	Description							
1	**stiffness reduction from 0% to 35% for the end of the forestay**							
	0	5	10	15	20	25	30	35
2	**stiffness reduction from 0% to 35% for the mid-span of the front beam**							
	0	5	10	15	20	25	30	35
3	**stiffness reduction from 0% to 35% for the top of the truss**							
	0	5	10	15	20	25	30	35

**Table 3:** The early warning results of FEVRD for different monitoring points in different time periods.

Monitoring Points	Threshold	Mean Value of FERVD for Monitoring Data in Different Time Periods						
		0 s~10 s	11 s~20 s	21 s~30 s	31 s~40 s	41 s~50 s	51 s~60 s	61 s~70 s
1	[0.0070~0.0389]	0.0159	0.0307	0.0312	0.0348	0.0342	0.0285	0.0279
2	[0.0115~0.0565]	0.0225	0.0183	0.0280	0.0327	0.0291	0.0245	0.0314
3	[0.0020~0.0681]	0.0350	0.0426	0.0280	0.0318	0.0381	0.0391	0.0317

## Data Availability

The data presented in this study are available upon request from the corresponding author.
